# Postnatal development of the electrophysiological properties of somatostatin interneurons in the anterior cingulate cortex of mice

**DOI:** 10.1038/srep28137

**Published:** 2016-06-20

**Authors:** Geng Pan, Jian-Ming Yang, Xing-Yue Hu, Xiao-Ming Li

**Affiliations:** 1 Department of Neurology, Brain Center, Sir Run Run Shaw Hospital, Zhejiang University School of Medicine, Hangzhou, Zhejiang, 310058 China; 2 Department of Neurobiology, Institute of Neuroscience, Key Laboratory of Medical Neurobiology of the Ministry of Health, Zhejiang Province Key Laboratory of Neurobiology, Collaborative Innovation Center for Brain Science, Zhejiang University School of Medicine, Hangzhou, Zhejiang, 310058 China; 3 Joint Institute for Genetics and Genome Medicine between Zhejiang University and University of Toronto, Zhejiang University, Hangzhou, Zhejiang, 310058 China

## Abstract

Somatostatin (SST)-positive interneurons in the anterior cingulate cortex (ACC) play important roles in neuronal diseases, memory and cognitive functions. However, their development in the ACC remains unclear. Using postnatal day 3 (P3) to P45 GIN mice, we found that most of the intrinsic membrane properties of SST interneurons in the ACC were developmentally mature after the second postnatal week and that the development of these neurons differed from that of parvalbumin (PV) interneurons in the prefrontal cortex. In addition, electrical coupling between SST interneurons appeared primarily between P12–14. The coupling probability plateaued at approximately P21–30, with a non-age-dependent development of coupling strength. The development of excitatory chemical afferents to SST interneurons occurred earlier than the development of inhibitory chemical afferents. Furthermore, eye closure attenuated the development of electrical coupling probability at P21–30 but had no effect on coupling strength. Eye closure also delayed the development of inhibitory chemical afferent frequency but had no effect on the excitatory chemical afferent amplitude, frequency or rise time. Our data suggest that SST interneurons in the ACC exhibit inherent developmental characteristics distinct from other interneuron subtypes, such as PV interneurons, and that some of these characteristics are subject to environmental regulation.

The medial prefrontal cortex (mPFC) consists of the interconnected anterior cingulate cortex (ACC), prelimbic cortex and infralimbic cortex. The ACC plays a role in regulating contextual fear memory^1^ and five-arm discrimination memory^2^. The ACC is also involved in spatial memory^3^, attention control^4^, emotion and motor functions^5^, Schizophrenia^6^ and epilepsy^7^,^8^. Furthermore, the ACC has been the focus of age-related error processing. During maturation, children, adolescents and young adults exhibit distinct neuronal activity in the ACC during an antisaccade task^9^.

The ACC contains approximately 20–30% interneurons^10^,^11^, and somatostatin (SST)-positive interneurons account for approximately 30% of these interneurons^12^. Cortical SST-positive interneurons arise from the medial ganglionic eminence^13^ as early as embryonic day 13.5^14^ and tangentially migrate across the developing cerebral cortex^15^,^16^. Chemical signaling inputs to SST interneurons are related to the inhibition and excitation (I/E) balance in the brain^17^,^18^. SST interneurons receive excitatory input from pyramidal neurons^19^ and afferents from interneurons, including vasoactive intestinal polypeptide^20^,^21^ and parvalbumin (PV)-positive^19^,^21^ interneurons. Such signals are synchronized^22^ and returned to the aforementioned neurons^19^,^21^, including PV-positive interneurons^23^. Mature SST interneurons control the input impact of pyramidal neurons primarily by preferentially innervating their distal dendrites^24^,^25^. This pyramidal neuronal control may require the participation of PV interneurons, which produce disinhibition by inhibiting SST interneurons^23^,^26^,^27^. Spikes of interneurons can be synchronized by electrical synapses/gap junctions that commonly interconnect two interneurons of the same type^23^.

SST interneurons play important roles in cognitive and neuronal disorders. In the hippocampus, SST interneurons facilitate the gating of hippocampal activity and thus contribute to epilepsy and schizophrenia; in addition, these interneurons participate in fear associated learning in the basolateral amygdala^26^,^27^. Recent evidence suggests that SST interneurons in the ACC are important for rewarded foraging tasks^28^ and sociosexual behavior^29^. Furthermore, both electrical coupling and GABA signaling in the ACC contribute to cingulate epilepsy^7^,^8^,^30^. However, the development of SST interneurons in the ACC, including their intrinsic properties, input chemical signals and electrical coupling, remains unclear. To address this issue, we used electrophysiology, including paired whole-cell recordings, to study the development of the electrophysiological properties of SST-positive interneurons.

## Results

### Postnatal development of the intrinsic membrane properties of SST-positive interneurons

**Figure 1:**
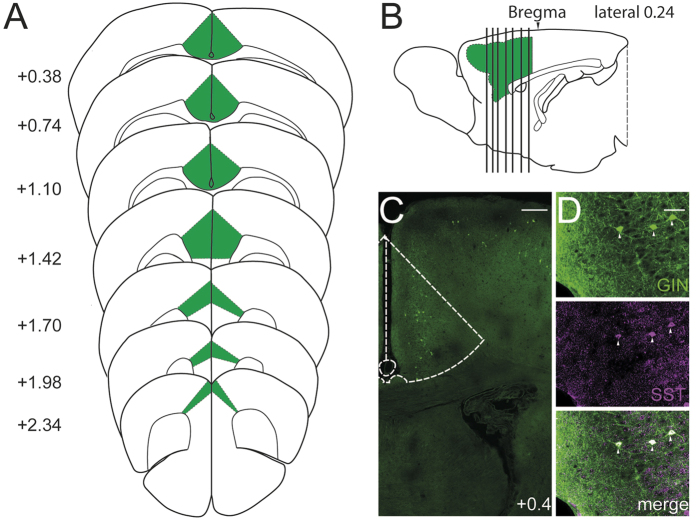
Cytoarchitecture of the mouse ACC. The ACC area used for the entire study. The (**A**) coronal plane is represented by the vertical bar in the (**B**) sagittal plane^33^. Panel (**C**) displays a brain slice from a GIN mouse, and the relative ACC area is indicated with a dotted line. The digit indicates the position of the brain slice relative to bregma (Unit: mm). Scale bar: 250 μm. Panel (**D**) shows the colocalization of GFP-positive and SST-positive cells. Scale bar: 50 μm.

**Figure 2:**
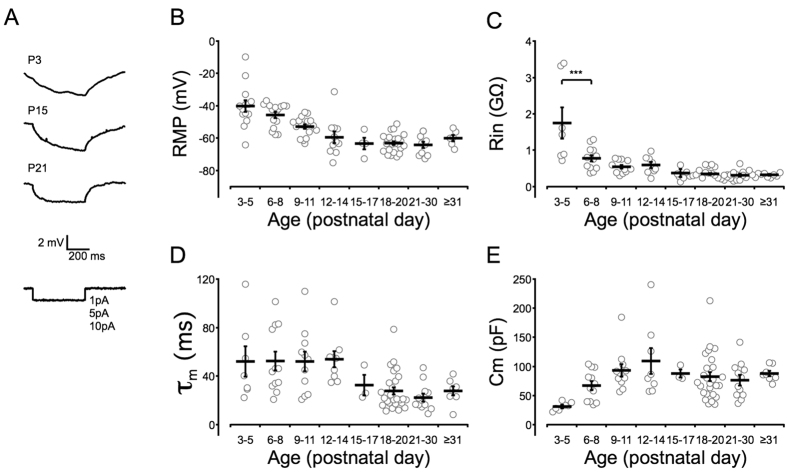
Development of the passive membrane properties of SST interneurons in the ACC after birth. Panel (**A**) indicates the voltage deflections used for measuring the passive membrane properties, including P3 (top), P15 (middle) and P21 (bottom) samples. Each trace is the average of 50 response curves. Panel (**B**) displays the resting membrane potential (RMP). Panel (**C**) displays the input resistance (R_in_) of cells, ***P < 0.001. Panel (**D**) shows the membrane time constant (τ_m_), and panel (**E**) shows the membrane capacitance (C_m_). The R_in_ significantly decreased within the first postnatal week, while the RMP and C_m_ gradually matured. The τ_m_ was stable prior to P14 and matured after P15; however, the maturation was slower than that of the other properties. Empty circles represent the results of eGFP-positive neurons from 44 mice. One-way ANOVAs with Tukey’s post-hoc tests were used.

**Figure 3:**
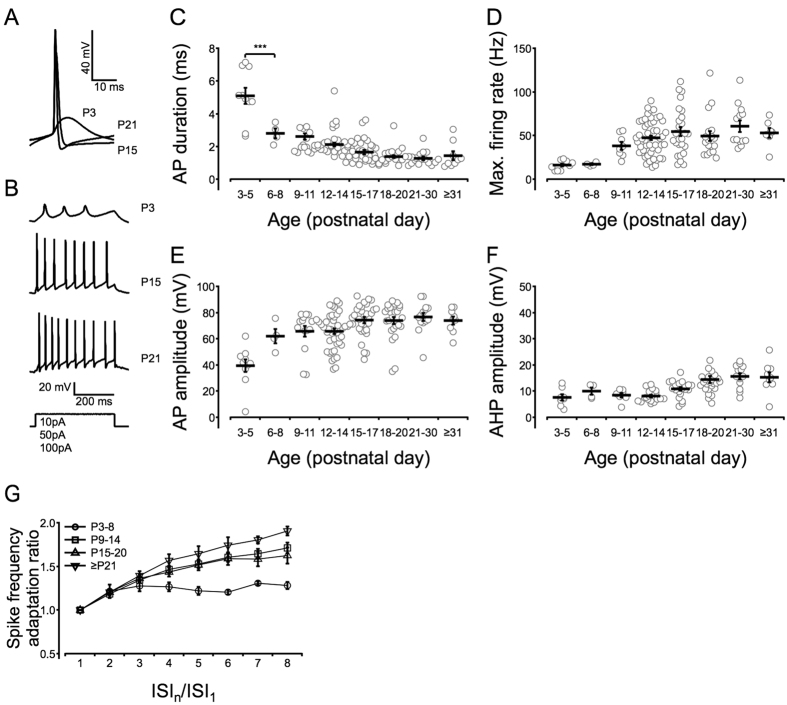
Development of the active membrane properties of SST interneurons in the ACC after birth. Panel (**A**) displays the single action potentials (APs) at P3, P15 and P21 which are evoked by suprathreshold depolarizing current (see “Methods” section). Panel (**B**) illustrates the firing patterns at the same ages as in panel (**A**). The injected currents for panel (**B**) is described in the “Methods” section. Generally applied currents depended on the age of the mice, i.e., 10–20 pA for P3–5, 20–50 pA for P6–8 and 50–200 pA for any age over P9. Panels (**C–F**) illustrate the developmental profiles of the AP duration, maximal firing rate, AP amplitude and after-hyperpolarization (AHP) amplitude, respectively. The methods used to obtain the membrane properties are listed in the “Methods” section. A significant decrease in AP duration was observed from P3–5 to P6–8, ***P < 0.001. The maximal firing rate and AP amplitude matured gradually with age, while the AHP amplitude exhibited a modest developmental change. Empty circles represent the results of eGFP-positive neurons from 71 mice. Panel (**G**) indicates the firing rate adaptation of difference ages. Resutls indicated that the mean adaptation ratio of older mice (≥P21) is larger than younger mice (P3–8) (for ISI_8_/ISI_1_). One-way ANOVAs with Tukey’s post-hoc tests were used.

In the neocortex of GIN mice, the overwhelming majority of eGFP-positive neurons are SST-positive GABAergic interneurons^31^,^32^. In the present study, we used sections of the ACC region (Fig. 1A–C), which has been defined in previous research^33^. We identified the colocalization of eGFP-positive and SST-positive cells in the ACC sections of GIN mice using SST antibodies (Fig. 1D). Unlike the cells in the barrel cortex, which are mostly Martinotti cells^32^,^34^, the morphology of the eGFP-positive interneurons in the ACC of GIN mice is not identical^31^. Therefore, to compare SST interneurons in the ACC with SST interneurons in the somatosensory cortex and PV interneurons in the prefrontal cortex, we studied their intrinsic electrophysiological properties, including passive properties [the resting membrane potential (RMP), input resistance (R_in_), membrane time constant (τ_m_) and membrane capacitance (C_m_), Fig. 2] and active properties [action potential (AP) duration, maximal firing rate, AP amplitude and after-hyperpolarization (AHP) amplitude, Fig. 3]. The results revealed that R_in_ and the AP duration changed rapidly within the first postnatal week and matured gradually during the second postnatal week (Figs 2C and 3C). The RMP, C_m_, maximal firing rate and AP amplitude matured gradually (Figs 2B,E and 3D,E), and the τ_m_ began to mature after P15 (Fig. 2D), while the AHP amplitude exhibited only a modest change (Fig. 3F). The most significant mean change was a 5.6-fold decrease in R_in_ (Fig. 2C). We also observed a 2.4-fold decrease in the τ_m_, a 3.4-fold increase in the C_m_ and a 1.6-fold decrease in the RMP (Fig. 2B,D,E). The maximal firing rate stabilized at ~50 Hz after P12–14 (Fig. 3D), whereas the AP duration decreased to 1–2 ms after postnatal week two, which was approximately one-third of the duration observed at P3–5 (Fig. 3C). As SST-positive interneurons are called low-threhold spiking cells^35^ and they display spike frequency adaptation, we then studied the values for the magnitude of adaptation as a function of age (Fig. 3G). Results indicated that the mean adaptation ratio of older mice (≥P21) is larger than younger mice (P3–8). The majority of the intrinsic properties of SST interneurons in the ACC and somatosensory cortex^34^ were similar; however, the developmental rate differed between SST and PV interneurons^36^.

### Development of electrical couplings between SST interneurons

**Figure 4:**
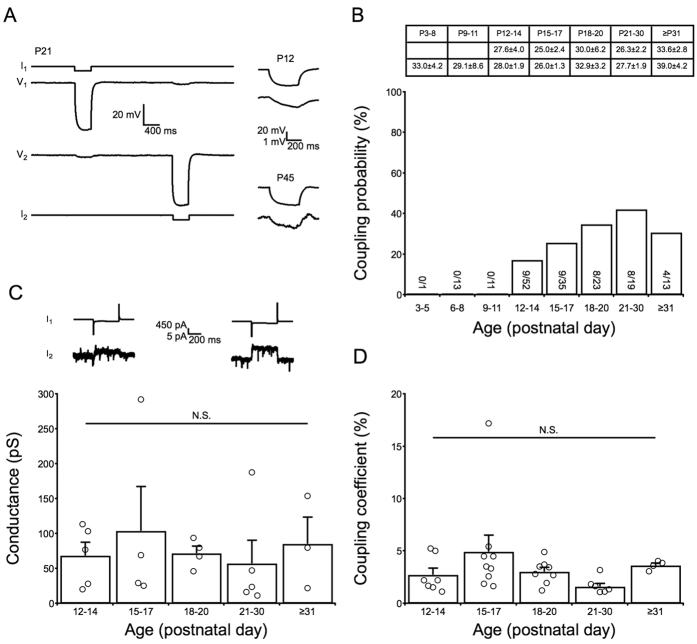
Development of electrical coupling between SST interneurons in the ACC. Panel (**A**) shows the electrical coupling at different ages: P12, P21 and P45. Cells were held at approximately −70 mV with current clamp methods. Voltage hyperpolarization (up to approximately −50 mV) was induced by current injection. Panel (**B**) illustrates the age-dependent electrical coupling that was observed until P21–30 (42%). This coupling decreased at ≥P31 (~31%) to a value similar to that at P18–20 (~35%). The table in the panel (**B**) indicates the inter-somatic distance of cells (unit: μm). The upper row illustrates distance between coupled cells and lower row illustrates distance between all paired recorded cells. There is no significant difference between each group. The number of pairs of cells for each age group is indicated at the bottom of the bar. The cells are from a total of 27 mice. Panels (**C,D**) show no developmental change in conductance (G_J_) or the coupling coefficient (CC). Two voltage-clamp examples used for measuring G_J_ are illustrated in panel (**C**). The detected values of I_2_ current were ~3 pA for P15 (left) and ~7 pA for P28 (right). One-way ANOVAs with Tukey’s post-hoc tests were used.

SST-positive interneurons are extensively interconnected by electrical couplings (also known as gap junctions) that synchronize SST-positive interneuronal activity^23^. Furthermore, gap junctions in the ACC mediate the synchronization of seizures^8^,^30^. Thus, studying the development of electrical couplings between SST-positive interneurons in the ACC is important for understanding disorders such as cingulate epilepsy. We injected hyperpolarizing current pulses into one cell and obtained paired whole-cell recordings. If the current induced a voltage deflection response in another cell, we considered the cells to be electrically coupled (Fig. 4A). The maximum distance between two recorded SST interneurons was 50 μm. From P3 to P11, it was difficult to detect electrical synaptic connections between SST interneurons (0 of 25 pairs) (Fig. 4B). At P12–14, electrical coupling appeared at an incidence of ~17%. Subsequently, the coupling incidence underwent an age-dependent increase up to ~42% at P21–30 (Fig. 4B). After P31, the coupling probability decreased to 31%, which was between the 26% observed at P15–17 and the 35% observed at P18–20 (Fig. 4B). As electrical coupling between interneurons is related to the intersomatic distance^37^, we collated the distance of paired cells we patched (Fig. 4B). There is no difference of intersomatic distance between each age groups. For electrically coupled cells, the inersomatic distance does not vary with age neither. The value for conductance (G_J_) was measured to estimate the electrical coupling strength. Surprisingly, G_J_ was not age-dependent. No significant difference was observed from P12–14 to ≥P31 (Fig. 4C). The mean G_J_ value detected was ~76 pS. A similar phenomenon was observed using the coupling coefficient (CC) (Fig. 4D) to measure electrical coupling strength, which also exhibited a non-age-dependent pattern and had a mean value of ~47%. This non-age-dependent development of electrical strength indicates that coupling probability and coupling strength develop independently.

### Development of chemical signaling inputs to SST interneurons

**Figure 5:**
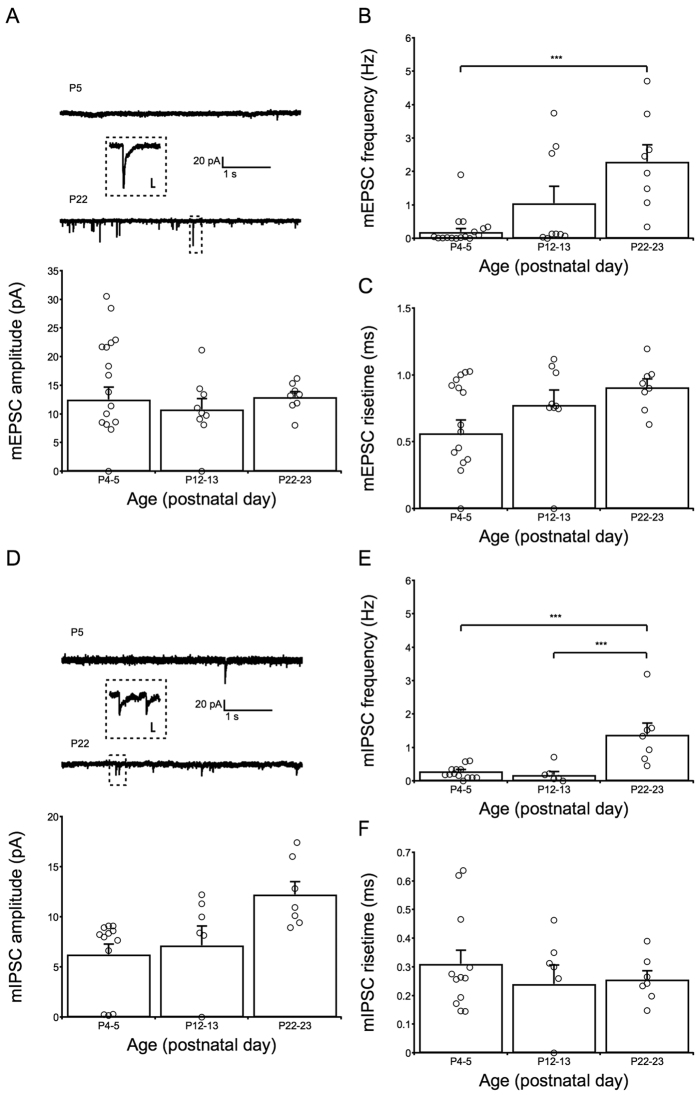
Detection of chemical inputs to SST interneurons in the ACC using miniature GABAergic/glutamatergic postsynaptic current (mI/EPSC) recordings. Panels (**A–C**) reveal the amplitude, frequency and rise time results obtained from mEPSC recordings (8 mice), respectively. Panels (**D–F**) reveal the amplitude, frequency and rise time obtained from mIPSC recordings (8 mice), respectively. Refer to the “Methods” section for details on the recording process. No age-dependent developmental changes in the amplitude and rise time of (**C**) glutamatergic or (**F**) GABAergic inputs were found. The frequency of (**B**) glutamatergic and (**E**) GABAergic inputs increased with age. The frequency of mEPSCs (**B**) was at a very low level on P4–5 but developed faster than the inhibitory inputs (**E**) because the frequencies of mIPSCs at P12–13 and P4–5 were both low. Scale bars in the dotted rectangle represent 10 pA (vertical bar) and 10 ms (horizontal bar). One-way ANOVAs with Tukey’s post-hoc tests were used.

SST interneurons receive afferents from interneurons, such as vasoactive intestinal polypeptide^20^,^21^ and PV-positive^19^,^21^ interneurons, and from pyramidal cells^19^. These signals are synchronized^22^ and returned to those neurons^19^,^21^,^23^. Chemical signaling inputs to SST interneurons are very important for the I/E balance in the brain^17^,^18^. Therefore, we examined the developmental profile of miniature glutamatergic postsynaptic currents (mEPSCs) and miniature GABAergic postsynaptic currents (mIPSCs) in SST interneurons (Fig. 5). For mEPSCs, both the amplitude and rise time matured early at P4–5 (Fig. 5A,C). The frequency of mEPSCs increased gradually and changed 11.3-fold from P4–5 to P22–23 (Fig. 5B). The amplitudes of both mIPSCs and mEPSCs displayed no significant differences from P4–5 to P22–23 (Fig. 5A,D). The rise time of mIPSCs matured as early as that of mEPSCs (Fig. 5F), although the absolute value was smaller (0.3 ms vs. 0.8 ms) (Fig. 5C,F). However, the frequency results indicated that the development of mIPSCs was slower than that of mEPSCs (Fig. 5B,E). The mIPSC frequency was low from P4–5 to P12–13 but significantly increased by 7.7-fold thereafter from P12–13 to P22–23 (Fig. 5E). No significant differences were found between P12–13 and P22–23 in the mEPSC frequency (Fig. 5B). In addition, we were able to detect neurons that did not express either mEPSC or mIPSC signals at P12–13.

### Effects of eye closure on electrical and chemical synapse signal development

**Figure 6:**
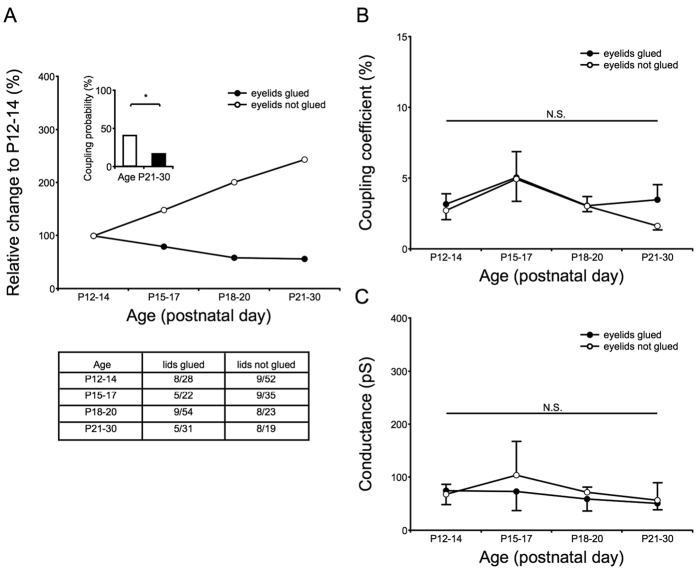
Effects of eye closure on electrical coupling incidence and coupling strength. (**A**) The eyelids of mice were glued at P10 (total of 21 mice), and electrical coupling incidence was then studied beginning at P12. The numbers of cell pairs with detected coupling and the total number of pairs studied for each age group are listed in the table of panel (**A**). Mice in which the eyelids were not glued had a coupling incidence of ~42% by P21–30. Eye closure attenuated this probability to ~16% (insert panel: *P = 0.046, Fisher’s exact test). Panels (**B**,**C**) show that eye closure did not affect coupling strength. Fisher’s exact tests and two-way ANOVAs with Tukey’s post-hoc tests were used.

**Figure 7:**
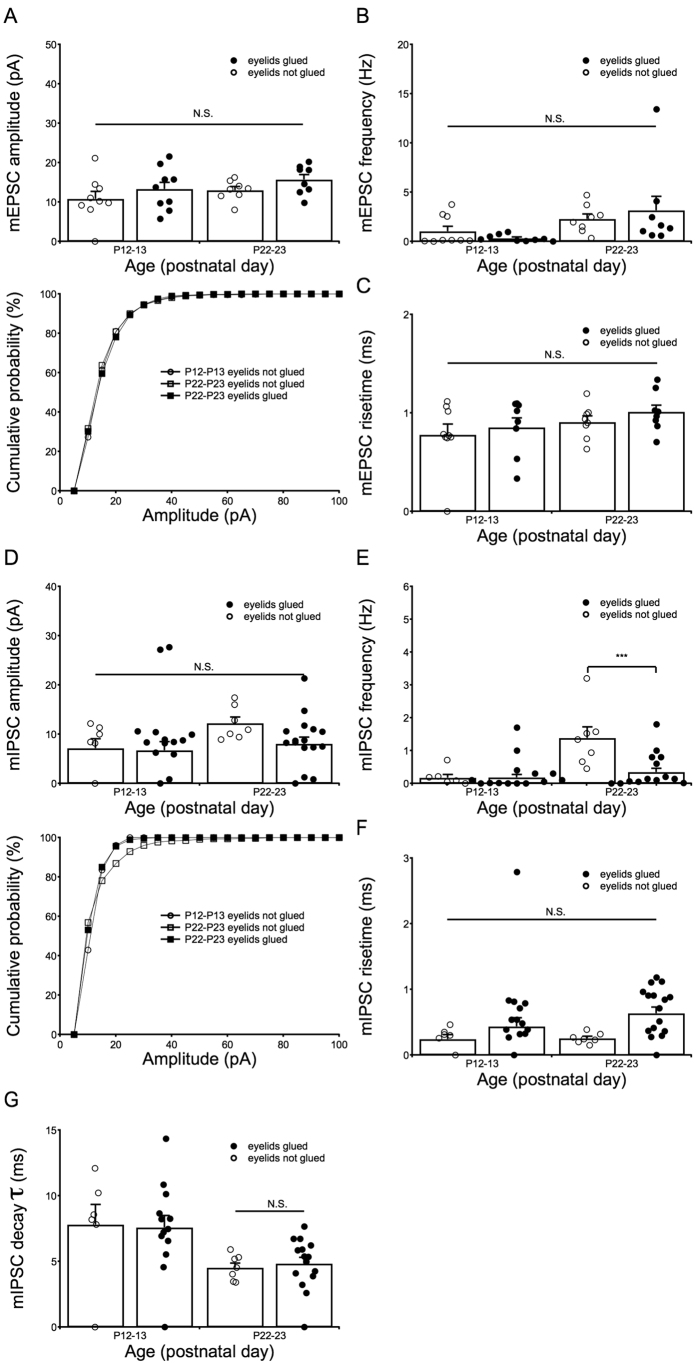
Effects of eye closure on the development of GABAergic and glutamatergic inputs to SST interneurons. After eye closure (4 mice for mEPSC recordings and 4 mice for mIPSC recordings), mI/EPSCs were compared at P12–13 and P22–23. Notably, (**A–C**) mEPSCs were not affected by eye closure. For mIPSCs, (**E**) only the development of frequency was strongly attenuated by eye closure; the frequency was maintained at a value similar to that observed at P12–13 (***P < 0.001). As the decay phase of mIPSC is related to the postnatal development, we collated the mIPSC decay time constant (decay τ). The mean value of the decay τ of younger mice (P12–13) is larger than the older mice (P22–23), but they are not statistically different (Fig. 7G). In addition, eyelids closure did not affect its development, either. Two-way ANOVAs with Tukey’s post-hoc tests were used.

Because rearing mice in darkness attenuates the development of the visual cortex^38^,^39^ and the ACC receives afferents from the visual cortex^40^, we examined whether the appearance of electrical synapses between SST interneurons was affected by eye closure. We glued the eyelids of P10 mice to delay the critical period^38^,^39^, a time window during which neural development can be shaped by experience^41^, but not to rewire the neuronal circuits in the visual cortex^42^,^43^. The eyelids of one to two littermates were not glued to verify that eye opening occurred at P14. Paired whole-cell recordings were completed with mice from P12 to P30. Based on both the G_J_ and CC values, no significant differences were observed in coupling strength (Fig. 6B,C). Notably, eye closure attenuated the development of coupling incidence during the first few days following eye opening (Fig. 6A). At P21–30, the coupling probability increased to 42% in the control mice; however, the probability remained at ~16% under eye-closure conditions (Fig. 6A insert panel). We also examined mI/EPSCs in mice during eye closure (Fig. 7). At P22–23, only the frequency of mIPSCs was delayed, remaining at the same frequency as that observed at P12–14 (Fig. 7E). These data suggest that the development of GABAergic neurons, but not glutamatergic neurons, was influenced by eye closure.

## Discussion

In this study, we focused on SST interneuronal development in the ACC of GIN mice from P3 to P45; the ACC region was defined based on previous research (Fig. 1)^33^. It should be noted that transgenic GIN mice have cortical eGFP-positive neurons that represent ~20% of the total SST-positive interneurons (see “Methods” section), and we do not know if the ~80% of SST-positive interneurons that are unlabeled may display different developmental properties.

Connections between interneurons can differ depending on the cortical area. For example, the probability of connection between PV and SST interneurons in the visual cortex is lower than that in other cortical areas^19^,^21^. However, it remains unclear whether the development of SST interneurons in the ACC differs from that in other areas. Therefore, we compared our results with those previously reported for SST interneurons in the somatosensory cortex^34^ and PV interneurons in the prefrontal cortex^36^.

Overall, the intrinsic properties of SST interneurons in the ACC displayed either a gradual maturation (RMP, τ_m_, C_m_, AP amplitude and maximal firing rate) or an abrupt maturation (R_in_ and AP duration), with the exception of the AHP amplitude, which exhibited a modest developmental change (Fig. 3F). Rapid maturation occurred during the first few days after birth, and most of the properties matured no later than P14, except for the τ_m_ which began to decrease after P15. Compared with the development of SST interneurons in the somatosensory cortex^34^, the R_in_ value obtained in our study was similar (~300 MΩ vs. ~250 MΩ), and the τ_m_ also matured at approximately P15. The AP duration was longer in the ACC than in the somatosensory cortex (1–1.2 ms vs. 0.8 ms), whereas the mature AHP amplitudes (~15 mV vs. ~14 mV) and maximal firing rates (40–60 Hz vs. 30–50 Hz with 100 pA stimulation) were nearly identical.

Compared with the development of PV interneurons in the prefrontal cortex^36^, certain passive intrinsic membrane properties of SST interneurons, including the τ_m_ and R_in_, matured ~2 days later, whereas active intrinsic membrane properties, such as the AP duration, matured ~6 days earlier. This finding suggests that distinct maturation mechanisms are utilized for different types of interneurons in the ACC. Because ambient GABA is related to vesicular GABA release^44^ and can regulate the formation of synapses^17^, the early maturation of the active membrane properties of SST interneurons may increase ambient GABA and further facilitate the development of neuronal circuits. The electrical coupling between SST interneurons (Fig. 4B) appeared at P12–14, which is later than that reported for PV interneurons (P5–6)^36^. The development of coupling strength between PV interneurons was found to be age-dependent, according to the G_J_ and CC values, which increased until P15–16 and then significantly decreased after P21 to levels similar to those observed at P5–8. However, this trend was not observed in the SST interneurons of the ACC. The G_J_ and CC values of these interneurons remained stable from the appearance of electrical coupling until over one month of age (Fig. 4C,D). This non-age-dependent development of the electrical coupling strength of SST interneurons in the ACC is consistent with previous research on the somatosensory cortex^45^. Evidence indicates that cellular C_m_ positively correlates with G_J_^46^. In accordance with our G_J_ results, we did not detect a decrease in C_m_ after P21. The electrical coupling strength of the SST interneurons was less than that of the PV interneurons, especially regarding G_J_ values (50–160 pS vs. 150–550 pS). Several factors regulate the G_J_ and CC amplitudes, e.g., the total number and location of gap junction channels, the G_J_ of a single gap junction channel and the membrane properties of coupled cells^47^. Because gap junctions are important for cortical signaling synchronization^8^,^30^,^48^,^49^ and cortical oscillation^50^, the late development of SST interneuronal electrical synapses indicates that SST interneurons may participate less than PV interneurons in cortical oscillation and signaling synchronization during the early postnatal weeks. The lack of electrical coupling before P11 may be due to that the neuronal processes and gap junctions of young and old mice are different. SST interneurons receive both excitatory inputs from pyramidal neurons^19^ and inhibitory inputs from various interneurons^19^,^20^,^21^; SST interneurons also participate in cortical synchrony^22^. Our results indicated that GABAergic inputs to the SST interneurons developed more slowly than the glutamatergic inputs during early postnatal development (Fig. 5B,E). This finding is in accordance with our previous report on PV interneurons^36^. The origin of GABAergic inputs to SST interneurons varies with brain region. PV interneurons rarely project to SST interneurons in the visual cortex^21^ but rather frequently connect to SST interneurons in other neocortical areas^19^. In the ACC, it remains unclear which type of interneuron first innervates the SST interneurons and whether the probabilities of different interneuronal couplings to SST interneurons vary among cortical areas.

Rearing mice in the dark delays the critical period and extends the immature period of the visual cortex^38^,^39^, an area that projects to the ACC via the medial subnetwork pathways^40^. Our results indicated that eye closure affects the development of SST interneuronal electrical couplings in the ACC (Fig. 6A). For rodents, the critical period of the brain is approximately 2–4 weeks after birth^34^, and the peak plasticity of the visual system occurs at approximately four weeks^51^. The effect of eye closure on electrical coupling probability was significant at P21–30 (Fig. 6A), which is close to the period of peak plasticity in the visual system. In addition to visual plasticity, there are other possible reasons. Altered circadian rhythms may regulate neuronal plasticity^52^. Eyelids closure may lead to decreased motor activity which is related to the neurogenesis^53^. Regarding chemical inputs, our results demonstrated that the development of mIPSC frequency was delayed (Fig. 7E). Additional studies are required to distinguish the types of interneurons that connect to SST interneurons in the ACC and to determine which developmental properties are affected by eye closure. Complex mechanisms for the developmental delay of electrical coupling and mIPSC frequency may exist because the incidence of electrical coupling and environmental chemical signaling may be mutually regulated^54^,^55^. Questions remain as to whether eye closure can decrease the electrical coupling probability between SST interneurons in mature brains and not just during the early postnatal period. Our findings provide additional evidence that earlier treatment improves the chances of infants with blinding eye diseases to avoid ACC-related developmental ailments. For example, blindness caused by retinopathy in premature infants can be cured/prevented by cryotherapy^56^; however, if treatment is delayed during the period of blindness, the development of neuronal circuits in the ACC can be adversely affected.

Dual eye closure and single eye closure have different physiological cascades. The suturing of a single eyelid during the critical period induces neuronal degeneration and circuit rewiring in the visual cortex^42^,^43^ due to imbalanced visual signaling inputs^41^. However, the closure of two eyes does not influence this balance^41^. Monocular deprivation has a stronger effect on the functional architecture of the visual cortex and may thus affect the electrical coupling incidence of SST interneurons in the ACC. The effect of monocular deprivation on the development of SST interneurons in the ACC warrants further study.

In conclusion, we systematically studied the developmental characteristics of SST-positive interneurons, including their intrinsic membrane properties, electrical couplings and chemical synapses. Our data revealed different maturation speeds for the membrane properties, a low electrical coupling incidence between SST interneurons during the first two postnatal weeks and a non-age-dependent coupling strength after the development of electrical coupling. In addition, we observed that the inhibitory chemical inputs matured more slowly than the excitatory chemical inputs did. All of our data suggest that immature SST interneurons might not participate in cortical oscillation and signaling synchrony during the first two weeks after birth. The data also illustrated the possibility of environmentally, rather than surgically, manipulating the development of the electrical coupling between SST interneurons and GABAergic inputs to SST interneurons in the ACC using eye closure. Attention should be paid to the risk of the abnormal development of interneuronal circuits in the ACC in infants recovering from blinding eye diseases, which could later lead to ACC-related neurological diseases.

## Methods

All animal-related experimental procedures were reviewed and approved by the Animal Advisory Committee at Zhejiang University in accordance with the National Institutes of Health Guidelines for the Care and Use of Laboratory Animals.

### Animals

Experiments were completed with GIN mice (*Mus musculus*) of either sex (#003718, Jackson Laboratory, Bar Harbor ME, USA). These mice express eGFP in a select group of SST-positive interneurons under the control of the *Gad1* promoter. In the neocortex, over 97% of the eGFP-positive neurons of GIN mice are SST-positive, and approximately 21.2% of all SST-positive neurons are labeled in GIN mice^31^,^32^.

### Slice preparation and maintenance solutions

In general, brain slices were prepared from P3 to P30 mice using Tris-based slicing and from P30 to P45 mice using NMDG-based slicing^57^. For the Tris method, anesthetized mice (ether inhalation) were perfused, and the brains were sliced using a microtome (VT1200S with Vibrocheck, Leica, Germany) with Tris artificial cerebrospinal fluid (aCSF) at 0–4 °C containing the following (in mmol/L): 76 Tris-HCl, 20 Tris base, 2.5 KCl, 1.2 NaH_2_PO_4_, 30 NaHCO_3_, 20 HEPES, 25 D-glucose, 5 Na-ascorbate, 2 thiourea, 3 Na-pyruvate, 10 MgSO_4_ and 0.5 CaCl_2_; the pH of the buffer was maintained at 7.35 with Tris base or HCl. Brain slices (300 μm) were maintained in oxygenated (95% O_2_/5% CO_2_) Tris aCSF at 22 °C for at least 30 min prior to the electrophysiological experiments. For the NMDG aCSF method, a cardiovascular perfusion was performed using ice-cold NMDG aCSF containing the following (in mmol/L): 93 NMDG, 93 NaCl, 2.5 KCl, 1.2 NaH_2_PO_4_, 30 NaHCO_3_, 20 HEPES, 25 D-glucose, 5 Na-ascorbate, 2 thiourea, 3 Na-pyruvate, 10 MgSO_4_ and 0.5 CaCl_2_; the pH of the buffer was maintained at 7.35 with NMDG or HCl. Brain slices were recovered in oxygenated NMDG aCSF at 34 °C for 10–12 min, followed by a secondary recovery in oxygenated HEPES aCSF at 22 °C for at least 60 min. The HEPES aCSF contained (in mmol/L) 92 NaCl, 30 NaHCO_3_, 25 D-glucose, 2.5 KCl, 1.2 NaH_2_PO_4_, 20 HEPES, 5 Na-ascorbate, 2 thiourea, 3 Na-pyruvate, 10 MgSO_4_, 0.5 CaCl_2_ and 12 N-acetyl-L-cysteine (NAC); the pH of the buffer was maintained at 7.35 with NaOH or HCl. The osmolality of each aCSF solution was maintained at ~310 mOsm.

### Electrophysiology

During each electrophysiology experiment, an individual brain slice was maintained in a recording chamber with the continuous perfusion of oxygenated standard recording aCSF (~1 mL/min), maintained at 32 ± 2 °C^36^, containing (in mmol/L) 124 NaCl, 2.5 KCl, 13 D-glucose, 24 NaHCO_3_, 1.2 NaH_2_PO_4_, 5 HEPES, 2 CaCl_2_, and 2 MgSO_4_; the pH was maintained at 7.35 with NaOH or HCl. Whole-cell patch-clamp recordings were performed using a Cl^−^ intracellular medium containing (in mmol/L) 110 K-gluconate, 40 KCl, 10 HEPES, 3 Mg-ATP, 0.5 Na_2_-GTP, 0.2 EGTA, with the pH maintained at 7.25 with KOH or HCl. Signals were acquired using a Digidata 1440A digitizer (Molecular Devices, Sunnyvale, CA) and amplifier (MultiClamp 700B, Molecular Devices) controlled by Clampex 10.4. Signals were filtered at 2 kHz for voltage clamp recordings or 3 kHz for current clamp recordings and then digitized at 10 kHz. Glass pipettes were made from borosilicate glass (with filament, Sutter, Novato, CA) with a resistance of 3.8–4.2 MΩ. Whole-cell patch-clamp recordings were performed after the formation of a gigaohm seal. The series resistance was not compensated. If the series resistance (maintained under 20 MΩ) increased over 30%, the recording was terminated.

We studied all the GFP-positive neurons in the ACC. However, the cortical GFP-positive neurons of GIN mice primarily locate in layer II/III and layer Va^32^ with fewer GFP-positive neurons in other layers. We observed a similar distribution in the ACC. Thus, the electrophysiological results mainly represent the neuronal properties of layers II/III and V.

For passive membrane properties, the RMP was measured within 3 min after establishing the whole-cell configuration. The R_in_ was obtained from voltage deflections (2–8 mV)^28^ under current-clamp conditions using the formula R_in_ = V/I. The τ_m_ was obtained by fitting a single exponential curve to the voltage deflection, and the C_m_ was calculated using the formula C_m_ = τ_m_/R_in_. To measure action potential properties, we applied small suprathreshold current steps to the cell from the RMP as previously reported^36^. The first spike trace evoked by a current step was used for the measurement of action potential properties. To measure the maximal firing rate, steps of depolarizing current were applied to the cell. Prior to the AP failure, the first interspike interval (ISI_1_) was used to calculate the maximal firing rate (Max. firing rate = 1/ISI_1_). AS we observed, the current was 10–20 pA for mice of P3–5, 20–50 pA for mice of P6–8and 50–200 pA for mice over P9. The AP threshold was defined as the point where dV/dt was 10 V/s^58^,^59^. The AP amplitude was defined as the voltage increase from the AP threshold to the AP peak. The AP duration was defined as the full width of time at the half-maximal amplitude. The AHP amplitude was defined as the voltage difference between the minimum value of the AHP and the AP threshold.

For electrical coupling detection, only mice with eyes that opened at P14 were used to reduce developmental diversity among individuals. One to two littermates were left intact to monitor eye opening times. For mice that underwent eye closure, the eyelids were glued at P10. We simultaneously patched two eGFP-positive cells (≤50 μm apart). The cellular membrane potential was held at −70 mV with a continuous current injection. Electrical coupling was evoked by applying a hyperpolarizing current (duration 500 ms, amplitude −10 to −300 pA) to one cell (ΔV_1_), and the ΔV_1_ was maintained at −50 mV^36^. If electrical coupling existed, a voltage deflection was observed in another cell (ΔV_2_). The CC was calculated as (ΔV_2_/ΔV_1_)*100. The threshold for electrical coupling confirmation was defined as 1% of the CC. For measuring the G_J_ of electrical coupling, one cell was hyperpolarized to −120 mV from −70 mV (400 ms) under voltage-clamp conditions. The gap junction-mediated current (ΔI_2_) was recorded in the other cell. The G_J_ was calculated as G_J_ = ΔI_2_/50 mV. Experiments were terminated if the whole-cell sealing was lost before recording the G_J_ or CC.

For the mEPSC recordings, 1 μM tetrodotoxin (#1078, Tocris Bioscience, Bristol, UK) and 100 μM picrotoxin (#1128, Tocris Bioscience) were added to the recording aCSF. For the mIPSC recordings, 1 μM tetrodotoxin, 50 μM AP-V (#3693, Tocris Bioscience) and 20 μM DNQX (#2312, Tocris Bioscience) were added to the recording aCSF, as previously described^36^. A threshold of 5 pA was used during the post-hoc analysis of the mI/EPSCs.

### Immunofluorescence staining

Anesthetized GIN mice were perfused with 4% paraformaldehyde (in phosphate-buffered saline, pH 7.4). Next, the brains were post-fixed for 24 h. Brain slices (50 μm) were prepared with a cryostat (CM3050, Leica). Brain sections were blocked with 3% donkey serum and 1% bovine serum albumin in Tris-buffered saline containing 0.5% Triton X-100 for 1 h at room temperature. The brain sections were then probed with mouse anti-SST (1:200, #ab103790, Abcam, Cambridge, MA, USA) at 4 °C for 72 h and visualized using an Alexa Fluor 647-conjugated donkey anti-mouse secondary antibody (1:200, #711605152, Jackson ImmunoResearch, West Grove, PA, USA).

### Fluorescence imaging

During the electrophysiology experiments, eGFP fluorescence was visualized with a mercury lamp mounted to an upright Nikon ECLIPSE FN1 (Tokyo, Japan). Confocal images were captured using the Olympus FV1000 system (Tokyo, Japan).

### Statistical analysis

Analysis methods and *P* values are indicated in the figure legends. The data were presented as the mean ± SEM. For comparisons between groups, two-way ANOVAs with Tukey post-hoc tests were used. For comparisons within a single group, one-way ANOVAs with Tukey’s post-hoc tests were used. Fisher’s exact test was used to compare electrical coupling probabilities. Significance levels of *P < 0.05, **P < 0.01 and ***P < 0.001 were used. The statistical analysis was performed using Igor Pro 6 and Prism 6. The electrophysiological data were analyzed offline with Igor Pro 6 (including Tarotools and Neuromatic). Confocal images were prepared with Fiji 2 and Adobe Illustrator CS 6.

## Additional Information

**How to cite this article**: Pan, G. *et al*. Postnatal development of the electrophysiological properties of somatostatin interneurons in the anterior cingulate cortex of mice. *Sci. Rep.* **6**, 28137; doi: 10.1038/srep28137 (2016).
